# Bifurcation functional significance score as predictor of mortality: a validating study

**DOI:** 10.1038/s41598-021-03815-6

**Published:** 2021-12-21

**Authors:** Dobrin Vassilev, Niya Mileva, Carlos Collet, Pavel Nikolov, Katerina Sokolova, Kiril Karamfiloff, Vladimir Naunov, Jeroen Sonck, Gianluca Rigatelli, Ghassan S. Kassab, Robert J. Gil

**Affiliations:** 1 Cardiology Department, Medical University Sofia, “Alexandrovska” University Hospital, Sofia, Bulgaria; 2 Cardiovascular Center OLV Ziekenhuis, Moorselbaan 164, 9300 Aalst, Belgium; 3 Department of Advanced Biomedical Sciences, University of Naples, Federico II, Naples, Italy; 4 Section of Cardiovascular Diagnosis and Endoluminal Interventions, Rovigo General Hospital, Rovigo, Italy; 5 California Medical Innovations Institute, San Diego, CA USA; 6 Mossakowski Medical Research Institute, Polish Academy of Science, Warsaw, Poland

**Keywords:** Interventional cardiology, Coronary artery disease and stable angina

## Abstract

Considerable progress has been made in the treatment of coronary bifurcation stenosis. Anatomical characteristics of the vessel and lesion, however, fail to give information about the functional significance of the bifurcation stenosis. To the best of our knowledge, there is no study that systematically establishes the baseline functional significance of coronary stenosis and its effect on procedural and clinical outcomes. Patients with significant angiographic bifurcation lesions defined as diameter stenosis > 50% in main vessel and/or side branch were included. FFR was performed in main vessel (MV) and side branch (SB) before and after percutaneous coronary intervention (PCI). 169 patients from Fiesta study (derivation cohort) and 555 patients from prospective bifurcation registry (clinical effect cohort) were analyzed to validate angiographic prediction score (BFSS) used to determine the potentially functional significance of coronary bifurcation stenosis. Bifurcation functional significance score (including the following parameters—SYNTAX ≥ 11, SB/MB BARI score, MV %DS ≥ 55%, main branch (MB) %DS ≥ 65%, lesion length ≥ 25 mm) with a maximum value of 11 was developed. A cut-off value of 6.0 was shown to give the best discriminatory ability—with accuracy 87% (sensitivity 77%, specificity 96%, p < 0.001). There was also a significant difference in all-cause mortality between patients with BFSS ≥ 6.0 vs. BFSS < 6.0–25.5% vs. 18.4%, log-rank p = 0.001 as well as cardiac mortality: BFSS ≥ 6.0 vs. BFSS < 6.0–17.7% vs. 14.5%, log-rank (p = 0.016). The cardiac mortality was significantly lower in patients with smaller absolute SB territory, p = 0.023. An angiographic score (BFSS) with good discriminatory ability to determine the functional significance of coronary bifurcation stenosis was developed. The value for BFSS ≥ 6.0 can be used as a discriminator to define groups with higher risk for all-cause and cardiac mortality. Also, we found that the smaller side branches pose greater mortality risk.

## Introduction

Considerable progress has been made in the treatment of coronary bifurcation stenosis^1^. Currently, we have increasing knowledge on how to achieve optimal flow dynamics in coronary bifurcation lesions. Several estimates of lesion and vessel significance, however, are highly subjective. The historical Medina classification is a simple tool used to describe the location of angiographically significant stenosis. It fails to give detailed anatomical information with a potential impact on functional significance (e.g., lesion length in main and side branches). Percutaneous coronary intervention (PCI) of functionally non-significant lesions will fail to provide clinical benefit and would expose the patient to unnecessary risk of periprocedural complications and mid-, long-term bleeding risk, because of intensive antiplatelet therapy^2–4^. To the best of our knowledge, insights in functional significance of bifurcation lesions *before* PCI are lacking. Available data focused on fractional flow reserve (FFR) measurements of residual side branch ostial stenosis significance *after* main vessel stenting^5,6^. No study systematically evaluated baseline functional lesion significance and its effect on procedural and clinical outcomes.

We previously determined the rates of functionally significant stenoses at the ostium of side branches after main vessel stenting, which correlated with changes on intracoronary electrocardiogram^7^. One surprising finding was the high rate of angiographically significant bifurcation stenoses, which were not functionally significant when measured with FFR. Therefore, we decided to extend recruitment of patients to confirm or reject our initial observations. The current study objectives were as follows: 1. To provide more complete information about frequency of functionally significant coronary bifurcation lesions before PCI (among patients with anatomically significant lesion, i.e. percentage diameter stenosis in main or side branch vessels); 2. To identify *anatomical* predictors of functional significance of coronary bifurcation stenosis. Based on those predictors to establish an angiographic score, which can be used for clinical application giving additional information about functional significance of stenosis and help in decision making; 3. Taking the developed score to examine in historical cohort of patients, what is probable frequency of functionally significant coronary bifurcation stenoses (defined according to developed score) and what is implication of this classification of patients on mortality.

## Methods

### Patient selection

**Figure 1:**
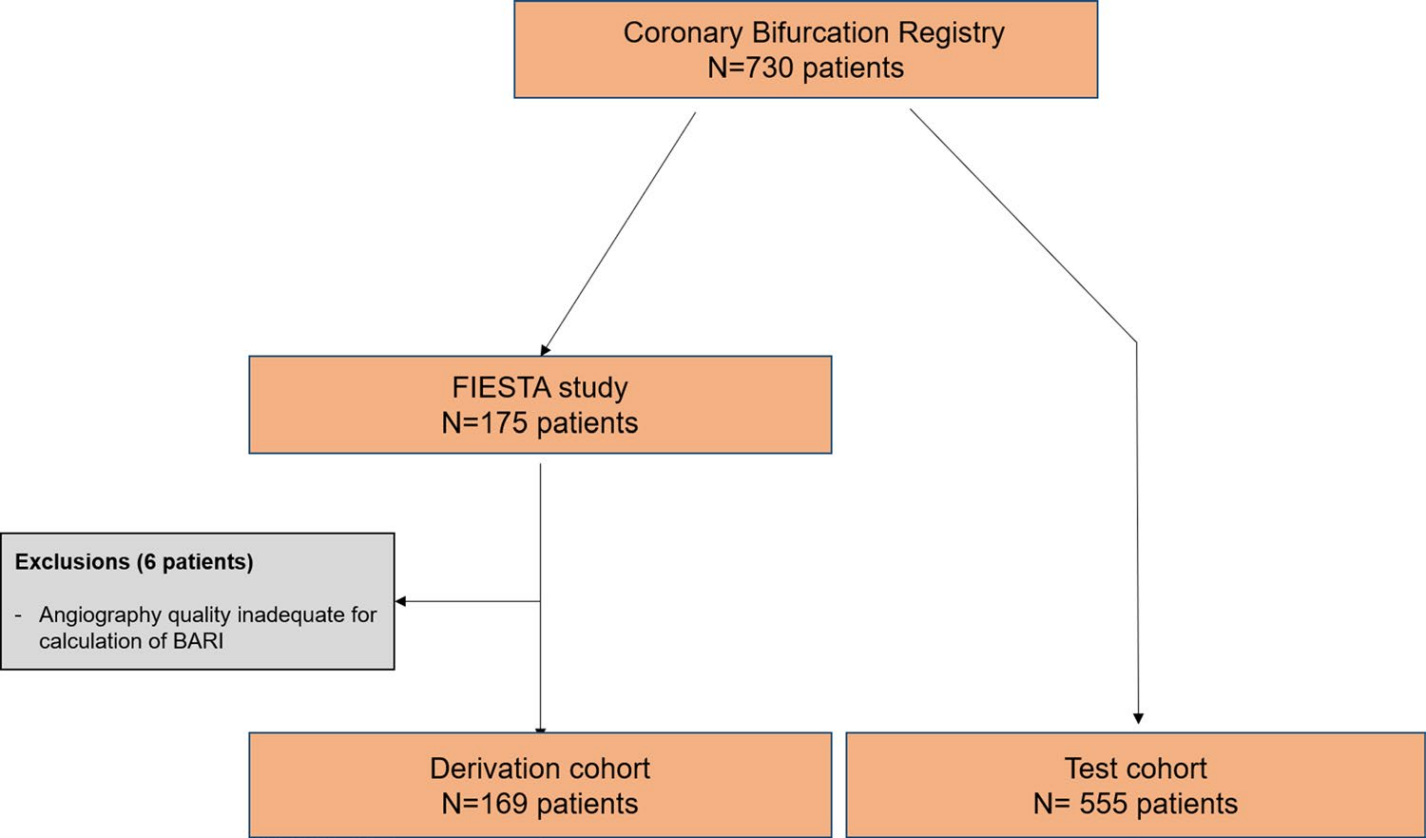
Patient selection flow chart.

All patients with coronary bifurcation lesions were included in an observational registry from July 2014. Patients included in the FIESTA study^7^ were used to form the derivation cohort. In general, these were patients with angiographic bifurcation lesions in a native coronary artery with diameter ≥ 2.5 mm and ≤ 4.5 mm and SB diameter ≥ 2.0 mm and percentage diameter stenosis > 50% in the main vessel. Patients with left main coronary artery stenosis, total occlusion before occurrence of the SB, lesion of interest located in an infarct-related artery, subjects with LVEF ≤ 30%, subjects with moderate or severe degree of valvular heart disease or primary cardiomyopathy were excluded. The data from the FIESTA study were used to extract variables predictive of functional significance of bifurcation lesions and to create a prediction score for functional significance (bifurcation functional significance score; BFSS). The remaining patients from the registry formed the clinical effect cohort for the score. These are patients with same inclusion criteria as patients in FIESTA group. Figure 1 illustrates the flow chart of patient selection.

### Definition of endpoints

A functionally significant bifurcation lesion was defined by the presence of a stenosis in the main vessel (MV) and/or side branch (SB) with FFR < 0.80 in accordance with the latest recommendations^8^. A main branch (MB) functionally significant lesion was considered a stenosis in the MV with an FFR measured distally from the bifurcation. Patients in both the derivation and clinical effect cohorts were followed up by telephone contact and/or clinical visit at 30 days and then monthly for vital status through their insurance number in the National Insurance Institute. In case of patient death, family members or family physicians were interrogated to define the cause of death. Cardiovascular death was defined as death with clearly determined cardiac origin or death from unknown reason. Myocardial infarction after hospitalization was diagnosed according to the Fourth definition of myocardial infarction^9^; i.e., as any rise in troponin or creatine-kinase MB more than 99th percentile of normal values in association with symptoms and/or documented ECG changes.

### Procedures

Initial FFR of MV and SB was performed using the PrimeWire or PrimeWire Prestige (Volcano Corp., USA). For all FFR measurements, intracoronary adenosine was given in increasing doses of 60 mcg, 120 mcg, and 240 mcg. A check for drift was performed before every measurement and at the end of the procedure. PCI was performed according to the current guidelines^10,11^. Provisional stenting was the default strategy in all patients. Pre-dilatation of MV was mandatory. After stenting and proximal optimization balloon inflation (left on operator discretion) FFR were measured in main and side branches. It was recommended that in case of SB FFR < 0.80, a balloon dilatation of SB should be performed. The SB was stented in case of TIMI flow less than 3, when visual diameter stenosis at ostium was more than 70%, despite kissing balloon inflation (KBI), and when the patient was symptomatic (i.e., with chest pain). If none of the above was present and FFR > 0.80, the SB was left untreated. Final KBI or sequential balloon inflation were performed at the discretion of the operator. All lesions were stented with second generation DES. Angiographic success was defined as the end procedural MV percent diameter stenosis (%DS) < 20% and SB stenosis < 70% without significant dissection nor flow impairment. Procedural success included angiographic success in the absence of in‐hospital MACE (death, stroke, and myocardial infarction). All patients received double antiplatelet therapy with aspirin and P2Y2 inhibitor. All procedures were carried out in accordance with the standard of clinical practice and regulations.

### Angiographic analysis

Dedicated bifurcation quantitative coronary angiography (QCA) analysis was performed according to the recommendation of the consensus on QCA methods for bifurcation lesions^12^. True bifurcation lesions were defined as visual percent diameter stenosis (%DS) > 50% at the SB. The minimal luminal diameter (MLD), reference vessel diameter (RVD) and %DS were measured for every segment of the bifurcation (i.e., proximal, and distal MV and SB) pre- and post-intervention. Lesion length was measured from proximal MV to distal MB (i.e., we considered beginning and ending points where hypothetically the stent will be implanted). SB lesion length was measured from the ostium to the first normal appearing part of the vessel. All analyses were performed by two investigators (P.N. and V.N) and in case of disagreement, a consensus was formed with additional analysis from the first author (D.V.). All the analyses were performed with dedicated General Electric QCA software and additionally with Micro Dicom QCA software. For the clinical effect cohort a visual angiographic analysis was performed from the same authors. For assessment of territory at risk and relative contribution of bifurcation lesion to all territory at risk adapted Bypass Angioplasty Revascularization Investigation Myocardial Jeopardy Index (BARI) score was calculated^17,18^.

### Statistical analysis

Differences between groups were examined with paired or unpaired t-tests as appropriate, with normal distributions. Otherwise, the Wilcoxon sign-ranked test and Mann–Whitney U-tests were used. Chi-square tests were applied for qualitative data. The area under the receiving operating characteristics curve (AUC) was used to assess the diagnostic accuracy of the test. Correlation analysis was performed (Pearson or Spearman test depending on type of data) between FFR values and possible predictors. Multivariate Cox regression analysis was performed for identification of independent predictors of all-cause death and cardiovascular death. Mortality rate in the two groups with functionally significant and non-significant bifurcation stenoses were compared.

#### Model development and description

##### First step

Univariate and then multiple logistic regression analysis were performed to identify independent predictors of functionally significant bifurcation lesion, as well as functionally significant stenoses in main branch and side branch directions. The quantitative predictors were dichotomized by performing receiver operator analysis (ROC) to identify the best discriminatory (highest accuracy) values associated with functionally significant stenosis in main and/or side branch. A second step logistic regression analysis was performed to confirm that the identified cut-off values are valid. Next, the model was internally validated performing bootstrapping. Then the regression coefficients were taken in ascendent order, with the smallest having a value of one and the next one—as ratio to the lowest. A ROC analysis was performed to assess model accuracy and to identify possible cut-off value, with the best discriminatory ability. To make model more practical the initial coefficients were round to nearest whole or half value and then the ROC analysis was performed again with the new values. If there was no statistical difference the value was accepted in that form, if not—the initial value was retained. A final internal validation with logistic regression analysis and bootstrapping was performed.

##### Second step

The model was applied to the population with coronary bifurcation stenoses, who were not assessed with FFR. Our assumption was that if the model can reliably predict the functional significance of bifurcation stenoses, the clinical outcomes in stented patients with potentially functionally significant and with potentially functionally non-significant stenoses should follow the pattern from DEFER and FAME one studies^2,4,13^.

The sample size was calculated assuming power of 80% and 95% confidence level, 16% cardiac mortality at 5 years in group with functionally significant stenosis and 10% event rate in patients with non-functionally significant stenosis (arithmetical mean between DEFER and FAME one populations), we would need a total sample of 490 patients to detect the same difference in cardiac mortality.

The study was investigator initiated, funded by the local institution (“Alexandrovska” University Hospital, Sofia, Bulgaria). The local ethics committee (Medical University of Sofia, Sofia, Bulgaria) approved the study and patients signed informed consent for participation into registry. All statistical calculations were performed via SPSS version 23 (SPSS, USA).

## Results

Derivation cohort: The final derivation cohort consisted of 169 patients (65% male). Flow chart of patient’s selection is shown in Fig. 1. Almost half had positive (FFR < 0.80) measurements in the MB (81/169, 48%), 28% (44/166) had FFR < 0.80 in both SB and MB. In only two patients a SB FFR of less than 0.80 was measured and decision for treatment was taken based on the large SB diameter. Lastly, in 3 patients it was impossible to measure FFR in the SB at baseline due to a tight ostial stenosis and/or steep angulation. The left ventricular ejection fraction (EF) overall was 58% ± 7% and was not different between functionally significant and non-significant groups. The differences in demographic characteristics and angiographic factors are presented in Table 1. LAD was the dominant vessel under investigation (81%, n = 137/169). Angiographically, patients with a positive FFR(< 0.80) had a more severe stenoses at any bifurcation segment with longer lesion length in the main vessel, but not in the SB. It is noteworthy that lesions with significant FFR had larger SB territories, but equal main branch areas at risk in comparison with non-significant functionally bifurcation lesions. It is also interesting, that functional significance depends on the length of the SB (reflected by SBBARI score) but not the diameter. On logistic regression analysis several factors were associated with functionally significant bifurcation lesion (Table 2).

**Table 1:** Patient’s demographic and angiography characteristics.

Patient characteristics	FFR ≤ 0.80 n = 81	FFR > 0.80 n = 88	P-value
Age (years)	65 ± 11	67 ± 10	0.163
Sex—males, n (%)	57 (71)	53 (60)	0.171
Hyperlipidemia, n (%)	74 (91)	84 (96)	0.142
Diabetes, n (%)	36 (45)	30 (34)	0.138
Renal failure, n (%)	24 (30)	25 (28)	0.818
Smoking, n (%)	45 (56)	37 (42)	0.089
Cerebrovascular disease, n (%)	13 (16)	11 (12)	0.477
Peripheral artery disease, n (%)	9 (11)	6 (7)	0.451
Previous myocardial infarction, n (%)	22 (27)	12 (14)	0.060
Previous PCI, n (%)	46 (57)	38 (43)	0.081
Beta blocker, n (%)	72 (89)	74 (84)	0.828
ACE inhibitor/ ARB, n (%)	71 (88)	74 (84)	0.868
Calcium antagonist, n (%)	45 (55)	36 (41)	0.086
Atrial fibrillation, n (%)	14 (17)	20 (23)	0.389
Angiographic parameters			
SYNTAX score	13 ± 4	7 ± 3	< 0.001
MV RVD, mm	3.32 ± .29	3.30 ± .45	0.757
MV %DS, %	61 ± 22	30 ± 20	0.000
MB RVD, mm	2.96 ± .23	2.83 ± 33	0.005
MB %DS, %	71 ± 13	35 ± 23	0.000
SB RVD, mm	2.43 ± .32	2.40 ± .36	0.586
SB %DS, %	56 ± 25	42 ± 24	0.001
Lesion length, mm	43 ± 20	18 ± 7	< 0.001
SB lesion length, mm	9 ± 10	10 ± 3	0.787
MB BARI score, %	30 ± 9	29 ± 7	0.677
SB BARI score, %	16 ± 6	13 ± 5	0.001
Multivessel disease, n (%)	42 (52)	29 (33)	0.017

All BARI score—percentage area at risk of left ventricle, based on all stenoses equal or more than 50% in diameter; MB BARI risk score—percentage area at risk supplied from a main branch of interest; SB BARI score—percentage area at risk supplied from a side branch.

**Table 2:** Independent predictors of functionally significant bifurcation stenosis (FFR ≤ 0.80) on multivariate analysis.

Predictor of all-cause mortality	OR	CI 95%	P-value
MV %DS	7.227	2.325–22.464	< 0.001
MB %DS	9.138	2.809–29.725	< 0.001
Lesion length	14.937	4.663–47.851	< 0.001
SYNTAX score	3.577	1.276–10.028	0.014
SBBARI/MBBARI	4.994	1.020–23.976	0.047

**Figure 2:**
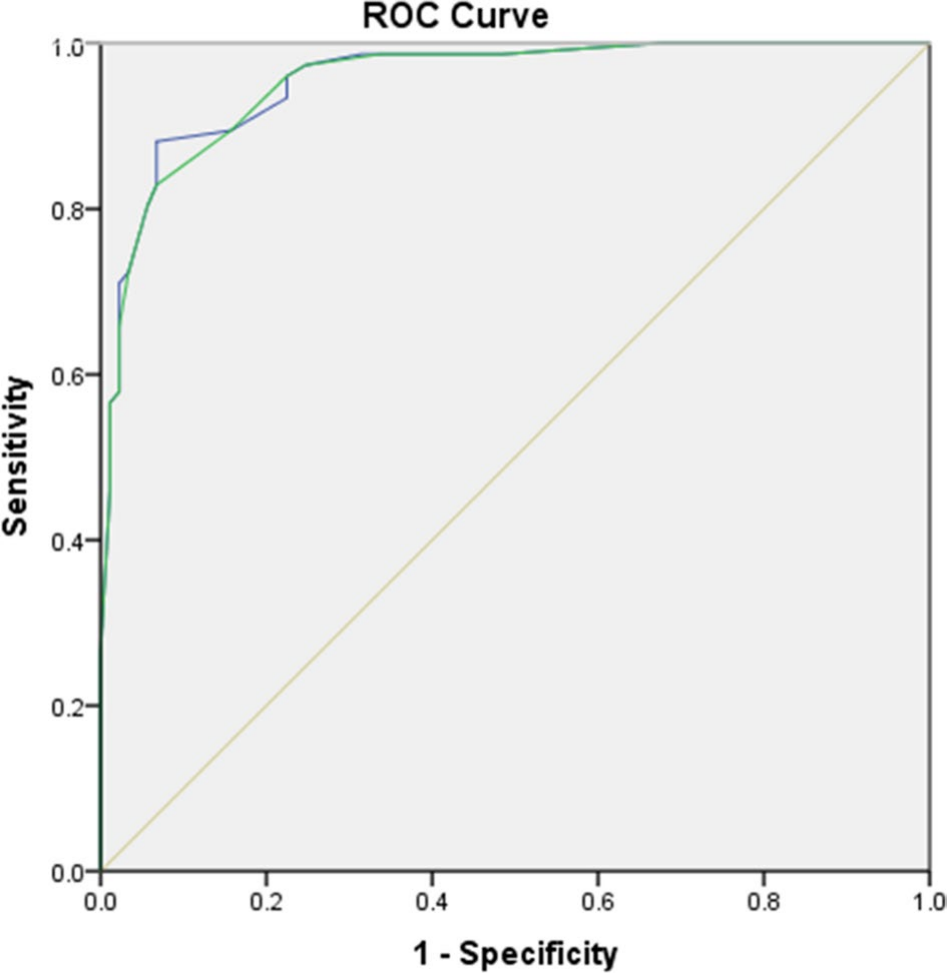
Receiver operator curves for initial and tuned model for prediction of coronary bifurcation stenosis functional significance. Blue line–initial model (AUC = .950, p < .001); green line–tuned model (AUC = .949, p < .001).

**Figure 3:**
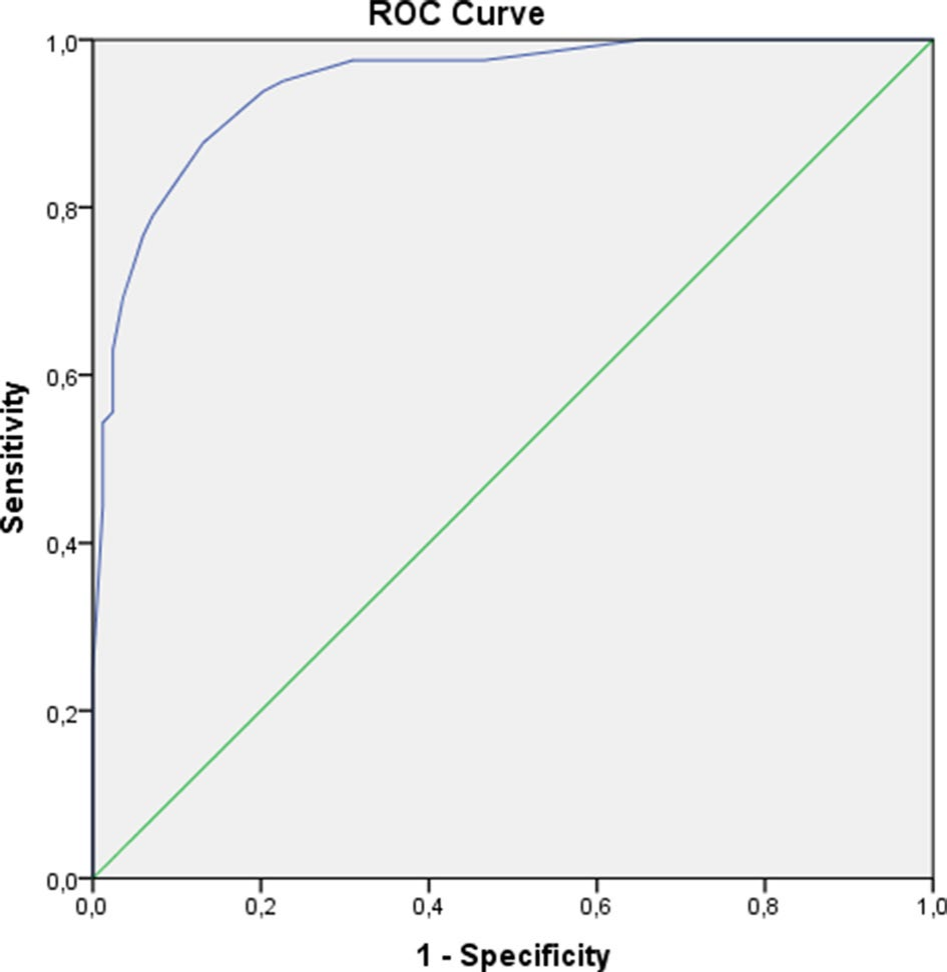
ROC curve analysis for the discriminatory ability of potential functional significant coronary bifurcation lesions. A cut-off value of BFSS of had 95% accuracy (sensitivity 77%, specificity 96%, p < 0.001. ROC–receiver operator’s curve; BFSS–bifurcation functional significance score.

To establish a model, we performed ROC analysis for identification of following cut-off values for the above independent predictors: MB %DS ≥ 65%, c-statistics 0.879 (CI 0.825–0.932, p < 0.001; sensitivity = 73%, specificity = 88%), MV %DS ≥ 55%, c-statistic 0.830 (CI 0.766–0.894, p < 0.001; sensitivity = 75%, specificity = 86%), lesion length ≥ 25 mm, c-statistic 0.898 (CI 0.849–0.947, p < 0.001; sensitivity = 86%, specificity = 74%), SYNTAX score ≥ 11, c-statistic 0.819 (CI 0.752–0.886, p < 0.001; sensitivity = 75%, specificity = 85%), ratio SBBARI/MBBARI ≥ 50%, c-statistic 0.600 (CI 0.516–0.687, p = 0.023; sensitivity = 52%, specificity = 58%). On basis of the above parameters with their relative weight in prediction of significant value of FFR, we developed an initial score, taking SYNTAX score > 11 with coefficient 1. Then we revaluated the model by increasing SBBARI/MBBARI ≥ 50% points to 1.5 (from 1.4 initially), and decreasing values for MB %DS ≥ 65% to 2.5 (from 2.55), rounding points for lesion length and MV%DS ≥ 5%to achieve the final version (Table 3, Fig. 2). The difference between the c-statistics of two ROC curves was only 0.001, which was not statistically significant. Next, we made internal validation by bootstrapping with χ^2^-test (p < 0.001 before and after bootstrapping) and logistic regression (beta = 3.935, CI 3.128–5.430, p < 0.001 before and after bootstrapping). The score had a maximum value of 11, with a significant difference between the groups with functionally significant and non-significant stenosis –9 ± 2 vs. 3 ± 2, p < 0.001. By ROC analysis, we determined a cut-off value of 6.0 that gave the best discriminatory ability with accuracy 87% (sensitivity 77%, specificity 96%, p < 0.001; Fig. 3).

**Table 3:** Bifurcation functional significance score (BFSS).

Parameter	Score
SYNTAX ≥ 11	1
SB BARI/MB BARI ≥ 50%	1.5
MV %DS ≥ 55%	2
MB %DS ≥ 65%	2.5
Lesion length ≥ 25 mm	4

Bifurcation Functional Significance Score (BFSS). Abbreviations same as Table 2. SB/MB BARI score—ratio of SBBARI score divided by MBBARI score.

Clinical effect cohort-distribution of potentially functionally significant coronary bifurcation lesions. The clinical effect cohort consisted of 555 patients followed-up for more than 6 months from index PCI. The differences between two groups are presented in Table 4. There were no significant differences in the risk factors rate between the two groups that underwent PCI. The frequency of LAD as a target vessel was significantly higher in the derivation than in the clinical effect cohort—80% vs 69%, p = 0.013. The two groups had similar sized vessels and degrees of stenosis, despite higher rate of multivessel disease in the clinical effect group. Those patients also had shorter lesions and smaller SB territories. The frequency of “true” bifurcation stenosis (side branch ostial stenosis more than 50%) was similar in the clinical effect group and stented group from the derivation cohort (63%, n = 348 and 60%, n = 53, respectively). The rates of SB stenting were non-significantly different between groups—24% (n = 20/81, from initially FFR < 0.80 patients) in derivation group vs. 18% (n = 100), as were rates of POT (73% vs. 69%, p = 0.309) and kissing balloon inflation (35%, n = 31 vs. 42%, n = 191, p = 0.531). In regard to the bifurcation functional significance score (BFSS), the patients in the clinical effect group had lower mean score in comparison with derivation group. In total 382 patients (69%) had a BFSS ≥ 6, meaning that with 87% accuracy those patients had functionally significant coronary bifurcation stenoses.

**Table 4:** Demographic and angiography characteristics of patients in derivation and clinical effect cohort.

Patient characteristics	Derivation cohort (n = 81)	Clinical effect cohort (n = 555)	P-value
Age (years)	65 ± 11	67 ± 10	0.337
Sex—males, n (%)	57 (71)	383 (69)	0.646
Hyperlipidemia, n (%)	74 (91)	522 (94)	0.162
Diabetes, n (%)	36 (45)	216 (39)	0.401
Renal failure, n (%)	24 (30)	172 (31)	0.941
Smoking, n (%)	45 (56)	222 (40)	0.054
Cerebrovascular disease, n (%)	13 (16)	83 (15)	0.661
Peripheral artery disease, n (%)	9 (11)	55 (10)	0.635
Previous myocardial infarction, n (%)	22 (27)	144 (26)	0.644
Previous PCI, n (%)	46 (57)	(48)	0.183
Beta blocker, n (%)	72 (89)	(88)	0.685
ACE inhibitor/ ARB, n (%)	71 (88)	(84)	0.578
Calcium antagonist, n (%)	45 (55)	(39)	0.019
Atrial fibrillation, n (%)	14 (17)	(22)	0.546
Angiographic parameters			
SYNTAX score	13 ± 4	12 ± 6	0.405
MV RVD, mm	3.32 ± .29	3.33 ± .43	0.421
MV %DS pre, %	61 ± 22	54 ± 31	0.103
MV %DS final, %	1 ± 6	2 ± 7	0.334
MB RVD, mm	2.96 ± .23	3.08 ± .75	0.593
MB %DS pre, %	71 ± 13	65 ± 27	0.090
MB %DS final, %	1 ± 4	2 ± 10	0.012
SB RVD, mm	2.43 ± .32	2.36 ± .74	0.561
SB %DS pre, %	56 ± 25	49 ± 32	0.062
SB %DS final, %	33 ± 33	28 ± 31	0.172
Lesion length, mm	43 ± 20	35 ± 20	0.004
MB BARI score, %	30 ± 9	29 ± 8	0.277
SB BARI score, %	16 ± 6	12 ± 60	0.001
Multivessel disease, n (%)	42 (52)	361 (65)	0.021
Bifurcation Functional Significance Score	9 ± 2	7 ± 3	0.001

### Clinical outcomes

All patients were followed-up for vital status. To be included in this analysis, at least 6 months follow-up following the PCI procedure was required. The mean follow-up time was 38 ± 18 months (median 40, IQR 23–55 months). For patients in the FIESTA registry, the all-cause mortality was numerically lower: 8.5% (n = 7/82) in the non-stent group and 12.6% in the stented group (n = 11/76; these includes patients not initially treated but receiving a stent in other institutions within 3 months from initial the procedure), p = 0.387. The cardiac mortality was also numerically lower, but statistically non-significantly different (9.8%, n = 8/82 vs. 11.5%, n = 10/88, p = 0.714). The all-cause mortality among patients in the clinical effect cohort was not significantly different in comparison with the stented cohort from the derivation cohort patients receiving a stent—15.9%, n = 88/555, p = 0.561; the same was true for cardiac mortality—13.7%, n = 76/555, p = 0.702 (Fig. 3). An additional analysis using BFSS as a continuous variable as well as dichotomous variable (BFSS lower or higher than 6) was performed (Tables 5 and 6).

**Table 5:** Independent predictors of all-cause mortality on multivariate analysis.

Predictor of all-cause mortality	HR	CI 95%	P-value	HR	CI 95%	P-value
Age	1.037	1.015–1.058	0.001	1.032	1.009–1.055	0.005
Diabetes	1.636	1.106–2.420	0.014	1.566	1.026–2.389	0.038
Symptoms (angina, SOB, both)	1.257	0.993–1.592	0.057	1.243	0.986–1.568	0.066
COPD	1.636	1.010–2.651	0.045	1.732	1.062–2.826	0.028
Mitral regurgitation > 1st degree	1.830	1.210–2.768	0.004	1.821	1.198–2.767	0.005
Dyslipidemia/statin	0.480	0.233–0.989	0.047	.483	0.233–.999	0.005
LBBB	1.613	0.946–2.751	0.079	1.624	0.945–2.792	0.079
Pre-PCI hsTnT > 0.010 ng/ml	1.803	0.987–3.294	0.055	1.912	1.048–3.490	0.035
BFSS ≥ 6	1.905	1.195–3.038	0.007			
BFSS (continuous variable)				1.048	0.971–1.131	0.097

*PCI* percutaneous coronary intervention, *hsTNT* high sensitive troponin T, *BFSS* bifurcation functional significance score.

**Table 6:** Independent predictors of cardiac mortality on multivariate analysis.

Predictor of cardiac mortality	HR	CI 95%	P-value	HR	CI 95%	P-value
Age	1.033	1.007–1.059	0.011	1.030	1.005–1.056	0.019
Dyslipidemia/statin	0.420	0.181–0.977	0.044	0.439	.189–1.020	0.056
Diabetes	2.349	1.443–3.823	0.001	2.430	1.496–3.950	< 0.001
Symptoms (typical and atypical angina)	1.340	1.023–1.756	0.033	1.345	1.027–1.761	0.031
LVPWT	1.195	1.048–1.363	0.008	1.201	1.053–1.369	0.006
Mitral regurgitation > 1^st^ degree	1.845	1.060–3.211	0.030	1.707	1.017–2.865	0.043
Pre-PCI hsTnT > 0.010 ng/ml	2.097	1.142–3.850	0.017	2.072	1.127–3.809	0.019
SBBARI score < 10%	1.757	1.091–2.833	0.020	1.802	1.105–2.941	0.018
BFSS ≥ 6	1.872	1.073–3.265	0.027			
BFSS (continuous variable)				1.093	0.996–1.199	0.060

*LVPWT* left ventricular posterior wall thickness, *PCI* percutaneous coronary intervention, *hsTNT* high sensitive troponin T, *BFSS* bifurcation functional significance score.

### Role of side branch territory for functional significance of coronary bifurcation stenosis and prognosis

**Figure 4:**
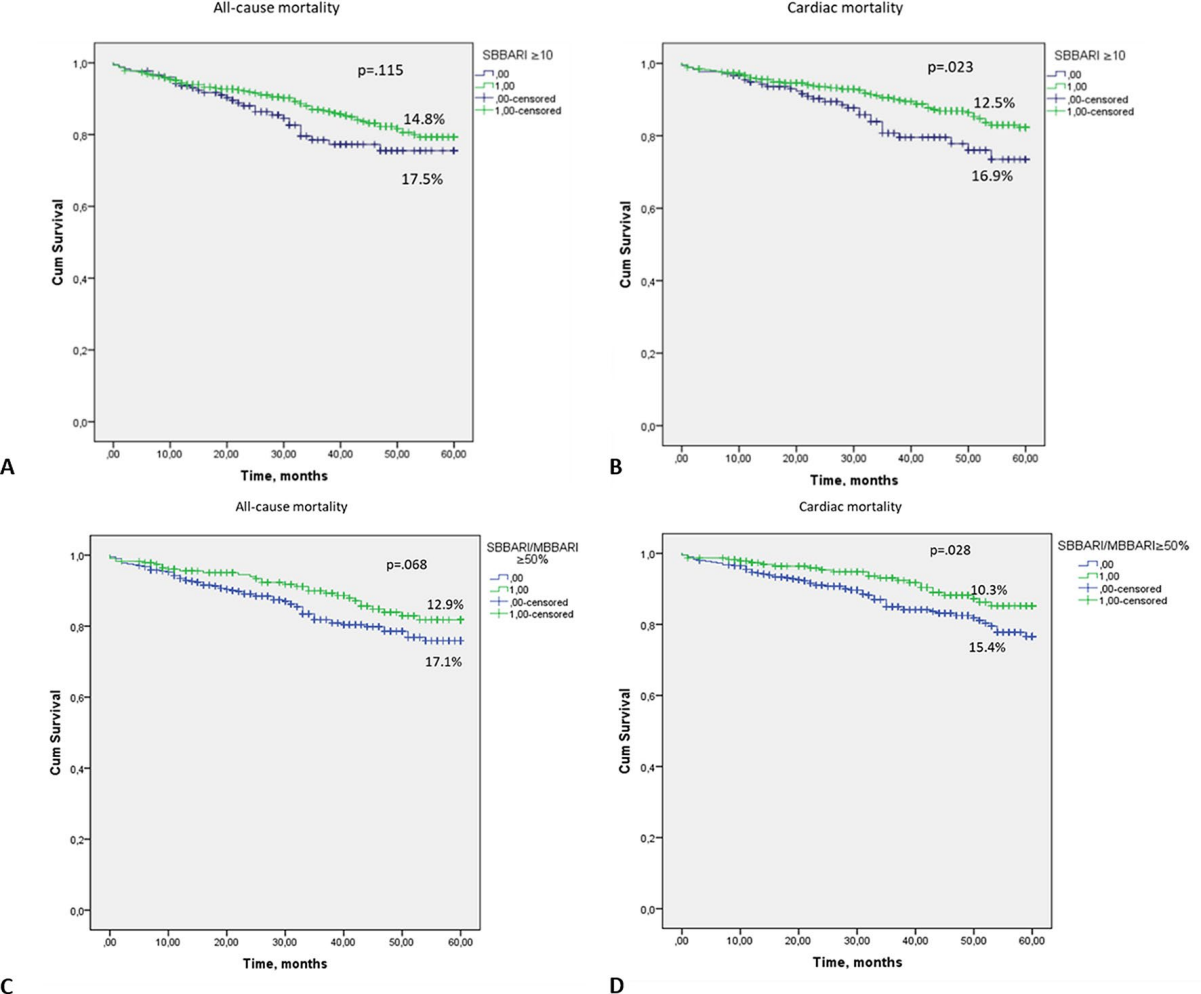
Kaplan–Meier curves: (**A**) All-cause mortality in patients with SBBARI score ≥ 10% and < 10%. (**B**) Cardiac mortality in patients with SBBARI score ≥ 10% and < 10%. (**C**) All-cause mortality in patients with SBBARI/MBBARI ≥ 50% and less < 50%; (**D**) Cardiac mortality in patients with SBBARI/MBBARI ≥ 50% and less < 50%.

We analyzed the impact of the absolute and relative side branch territory size on the functional significance of bifurcation stenosis and mortality. On ROC analysis (c = 0.612, p = 0.032) the SBBARI ≥ 12% had a 75% sensitivity and 52% specificity to detect functionally significant bifurcation stenosis. If the cut-off of the SB territory at risk was set at 10% (the traditional value for territory at risk requiring revascularization) the sensitivity increase to 92%, but with extremely low specificity of 29%. There was no relation between SB reference diameter and bifurcation stenosis functional significance. Regarding the risk of death, there was a significant difference in cardiac mortality with values of SBBARI starting from 10% (Fig. 4). The cardiac mortality was significantly *lower* in patients with smaller absolute SB territory—p = 0.023. The same was true for relative SB territory—a larger SBBARI/MBBARI ratio was related with numerically lower all-cause mortality and statistically significantly lower cardiac death. There was also a significant difference in all-cause mortality between patients with BFSS ≥ 6 vs. BFSS < 6–25.5% vs. 18.4%, log-rank p = 0.001, as well as cardiac mortality: BFSS ≥ 6 vs. BFSS < 6–17.7% vs. 14.5%, log-rank p = 0.016. Thus, the BFSS, which incorporates relative side branch territory, has a high discriminatory ability to select patients at risk of death. The cardiac mortality was significantly lower in patients with smaller absolute SB territory, p = 0.023.

These numbers are higher than expected from historical data, probably, because in the previous studies bifurcation lesions were not included. Thus, BFSS not only demonstrated high accuracy in prediction of FFR significant coronary bifurcation lesions, but also demonstrated very good discriminatory ability to differentiate patients with high risk of death (both all-cause and cardiac).

## Discussion

There are several new findings in our study: First, an angiographic score (BFSS), which can predict functional significance of coronary bifurcation lesions with reasonable accuracy (> 85%) was developed. The variables forming the score were not related to the bifurcation only, but also considering the overall disease severity and territory supplied by MB and SB. The score was internally validated with bootstrapping, demonstrating very high discriminatory ability. At follow-up the deferred from PCI group had the same rates of survival, giving additional assurance about the findings. The BFSS is easy to calculate—it requires only usual QCA on main vessel lesion and visual comparison of lengths of MB and SB (the relative side branch territory). Second, we estimated a proportion of patients with functionally significant bifurcation stenoses and explored the rates of mortality among those patients based on BFSS values. The frequency of probably functionally significant bifurcation stenoses estimated by BFSS ≥ 6 was around 2/3 of whole examined population and if we consider 85% accuracy of our score, then 57% of patients would be with FFR < 0.80. The value for BFSS ≥ 6 was also with best discrimination ability in all-cause and cardiac mortalities. Overall, there is a high probability that at the time of PCI between 20–40% of the patients will have borderline functional significance bifurcation stenosis. This confirms our earlier observation from FIESTA study^7^. We evaluated the clinical applicability of BFSS ≥ 6 instead of real performance of FFR in our clinical effect cohort. It appeared that this cut-off value could not only predict functional significance of coronary bifurcation lesion, but also to give prognostic information about future mortality. Our results are similar to those in DEFER study, where patients with significant (FFR < 0.75) stenoses were treated together with patients with FFR > 0.75, randomized to PCI—our patients in clinical effect cohort, with potentially functionally non-significant stenoses (BFSS < 6) had statistically significantly lower mortality rates. The larger actual sample size and higher number of events could explain significance of observed difference, despite that it was smaller than expected during statistics calculations. That result should be further evaluated in larger study.

Finally, for the first time, we determined the role of SB territory in relation to mortality in patients with stented coronary bifurcation stenoses. An unexpected finding was that the smaller the absolute and relative territory supplied by the side branches, the greater mortality risk they pose. Other previously validated angiographic risk score as SYNTAX and BARI risk scores are useful tools, that can be used in every catheterization laboratory and does not require any specific equipment or software for calculation^14–16^. Not unexpectedly, larger side branches, with a larger absolute and relative territory were more frequently associated with FFR < 0.80. It is interesting that the ratio of SB and MB vessel diameter also correlated with functional significance but was not independently related with FFR < 0.80. The possible explanation is that diffuse disease precludes adequate assessment of vessel sizes and its relation to myocardial mass, especially when assessed with coronary angiography^17^. Calculation of absolute SB territory (SB BARI score) requires a high-quality angiogram, including a good visualization of the length of every coronary artery branch with a diameter more than 1 mm. The SBBARI/MBBARI ratio is dimensionless, does not require visualization of the whole coronary artery tree and is easy to apply in every patient. Our data could not be directly compared with previous studies on this topic as they included patients with left main stenosis, as well as completely normal bifurcations, assessed with computer tomography^18,19^. The major advantage of our method is the ease of use and applicability in daily practice.

There is a general belief that larger side branches have higher impact on patient prognosis. One speculation is that smaller SBs are associated with larger main branches, supplying larger myocardial territory and in case of stent-related event, the fatality rate would be higher. There was no statistically significant difference between MBBARI scores in groups with smaller of larger SB territories. Another possible explanation is that larger side branches give a better collateral supply to a neighbor territory and in case of event in main branch, the SB could ensure a minimum supply and limit possible myocardial necrosis, thus improving patient prognosis. This hypothesis deserves further investigation.

The frequency of all-cause and cardiac death in our population is higher than reported in previous studies^20,21^. We could not confirm the data published previously, that periprocedural increase in troponin is associated with higher mortality risk^22,23^. We tested different levels of increase in postprocedural troponin values (up to 70xUNL) for its association with mortality, but neither was significant. However, the baseline concentration of troponin was related with outcome and the cut-off value associated with mortality was *below* the currently accepted upper normal limit for high-sensitivity troponin (UNL < 0.012 ng/ml; the value in our study related with increase in death is 0.010 ng/ml). This raises question about the baseline risk stratification and current reference standards, especially for the patients with complex coronary artery disease. From multivariate analysis, we did not observe any effect on mortality of side branch compromise (diameter stenosis more than 50% after stenting main vessel), SB stenting, type of pre- or post-dilatation, as well as number of stents or total stent length, despite that each of these factors were univariately associated with mortality rates.

## Conclusion

An angiographic score (BFSS) with good discriminatory ability to determine the potential functional significance of coronary bifurcation stenosis was validated. The value for BFSS ≥ 8.5 can be used as discriminator to define groups with higher risk for all-cause and cardiac mortality. Also, our analysis showed that the smaller as an absolute and relative territory side branches pose greater mortality risk.
